# Supplementary material for: Construction and validation of a 15-gene ferroptosis signature in lung adenocarcinoma
Source: PeerJ. 2021 Jul 7;9:e11687. doi: 10.7717/peerj.11687 (PMC8272465; doi:10.7717/peerj.11687)
Supplement: Supplemental Information 11 — (A) The PPI network analyzed by STRING database and visualized in Cytoscape software. (B) The correlation network of the differentially expressed ferroptosis-related genes. In the correlation network, the correlation coefficients are represented by different colors. PPI, protein-protein interaction; STRING, Search Tool for the Retrieval of Interacting Genes. [file peerj-09-11687-s011.pdf]

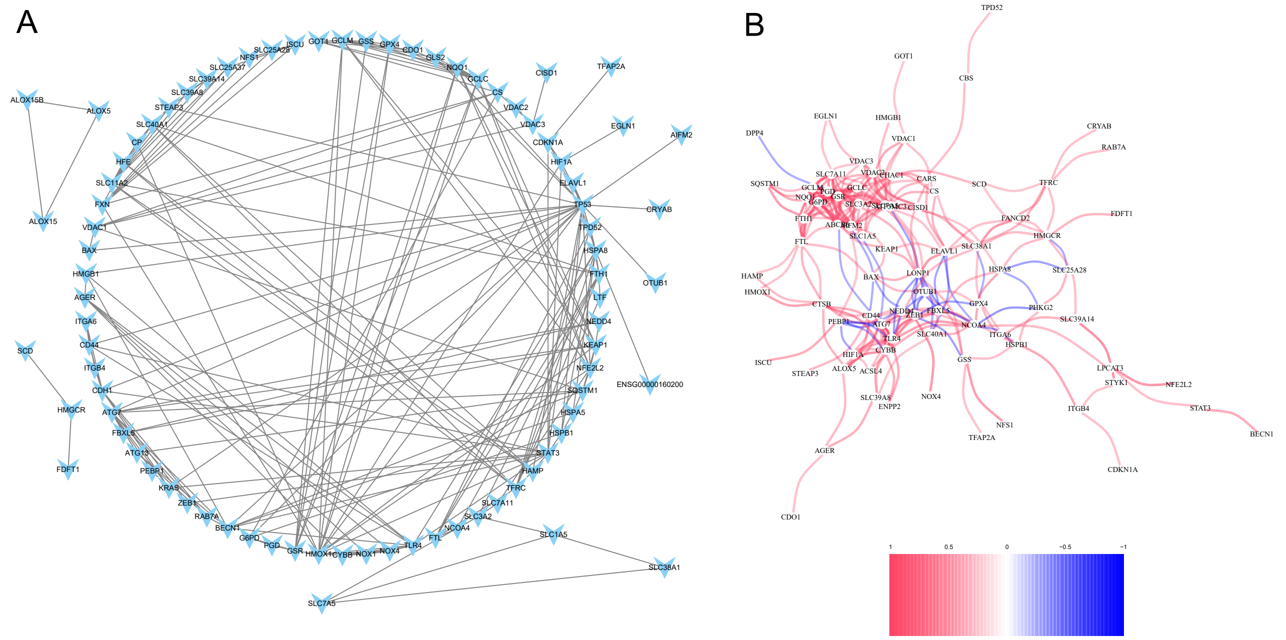
A
ALOX15B
ALOX5
ALOX15
SCD
HMGCR
FDFT1
GOT1
GCLM
GSS
GPX4
CDO1
GLS2
NQO1
GCLC
CS
VDAC2
VDAC3
CDKN1A
HIF1A
ELAVL1
TP53
TPD52
HSPA8
FTH1
LTF
NEDD4
KEAP1
NFE2L2
SQSTM1
HSPA5
HSPB1
STAT3
HAMP
TFRC
SLC7A11
SLC3A2
NCOA4
FTL
TLR4
NOX4
NOX1
CYBB
HMOX1
GSR
PGD
G6PD
BECN1
RAB7A
ZEB1
KRAS
PEBP1
ATG13
FBXL5
ATG7
CDH1
ITGB4
CD44
ITGA6
AGER
HMGB1
BAX
VDAC1
FXN
SLC11A2
HFE
CP
SLC40A1
STEAP3
SLC39A8
SLC39A14
SLC25A37
NFS1
SLC25A28
ISCU
CISD1
TFAP2A
EGLN1
AIFM2
CRYAB
OTUB1
ENSG00000160200
SLC1A5
SLC38A1
SLC7A5
B
TPD52
GOT1
CBS
EGLN1
HMGB1
VDAC1
DPP4
VDAC3
SLC7A11
VDAC2
CHAC1
CARS
SCD
TFRC
CRYAB
RAB7A
SQSTM1
GCLM
GCLC
CS
NQO1
PGD
GSR
G6PD
SLC3A2
CISD1
FTH1
ABCB1
AIFM2
SLC1A5
FANCD2
FDFT1
FTL
KEAP1
ELAVL1
SLC38A1
HMGCR
HAMP
BAX
LONP1
HSPA8
SLC25A28
HMOX1
OTUB1
CTSB
GPX4
PHKG2
CD44
NEDD4
FBXL5
ZEB1
SLC39A14
PEBP1
ATG7
NCOA4
SLC40A1
ITGA6
TLR4
HSPB1
HIF1A
CYBB
ISCU
GSS
ALOX5
ACSL4
LPCAT3
STEAP3
STYK1
NFE2L2
SLC39A8
NOX4
ENPP2
ITGB4
STAT3
NFS1
BECN1
TFAP2A
AGER
CDKN1A
CDO1
1
0.5
0
−0.5
−1
