# Supplementary figures and images for: Construction and validation of a 15-gene ferroptosis signature in lung adenocarcinoma
Source: PeerJ. 2021 Jul 7;9:e11687. doi: 10.7717/peerj.11687 (PMC8272465; doi:10.7717/peerj.11687)

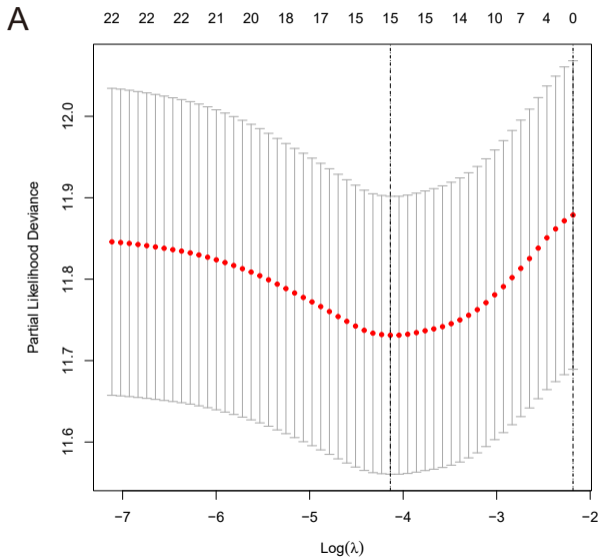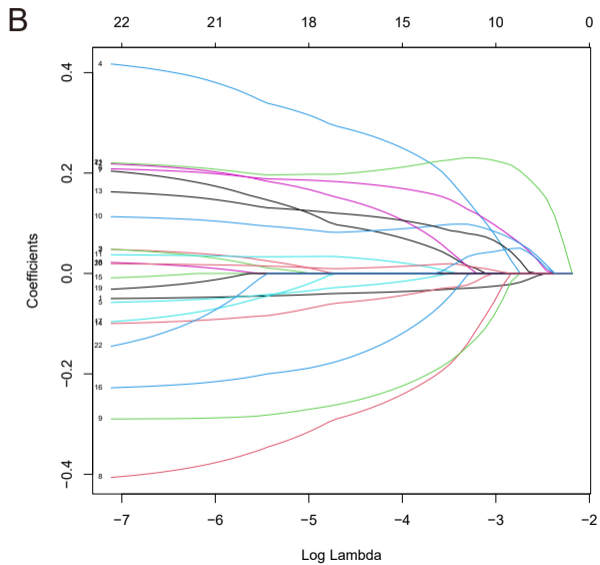

Supplement: Supplemental Information 12 — (A) Identification of the penalty parameter (λ) in the LASSO algorithm via 10-fold cross-validation; (B) LASSO coefficient profiles of the 22 candidate genes. The dotted vertical lines are plotted at the optimal values following the minimum criteria (left) and “one standard error” criteria (right). [file peerj-09-11687-s012.pdf]

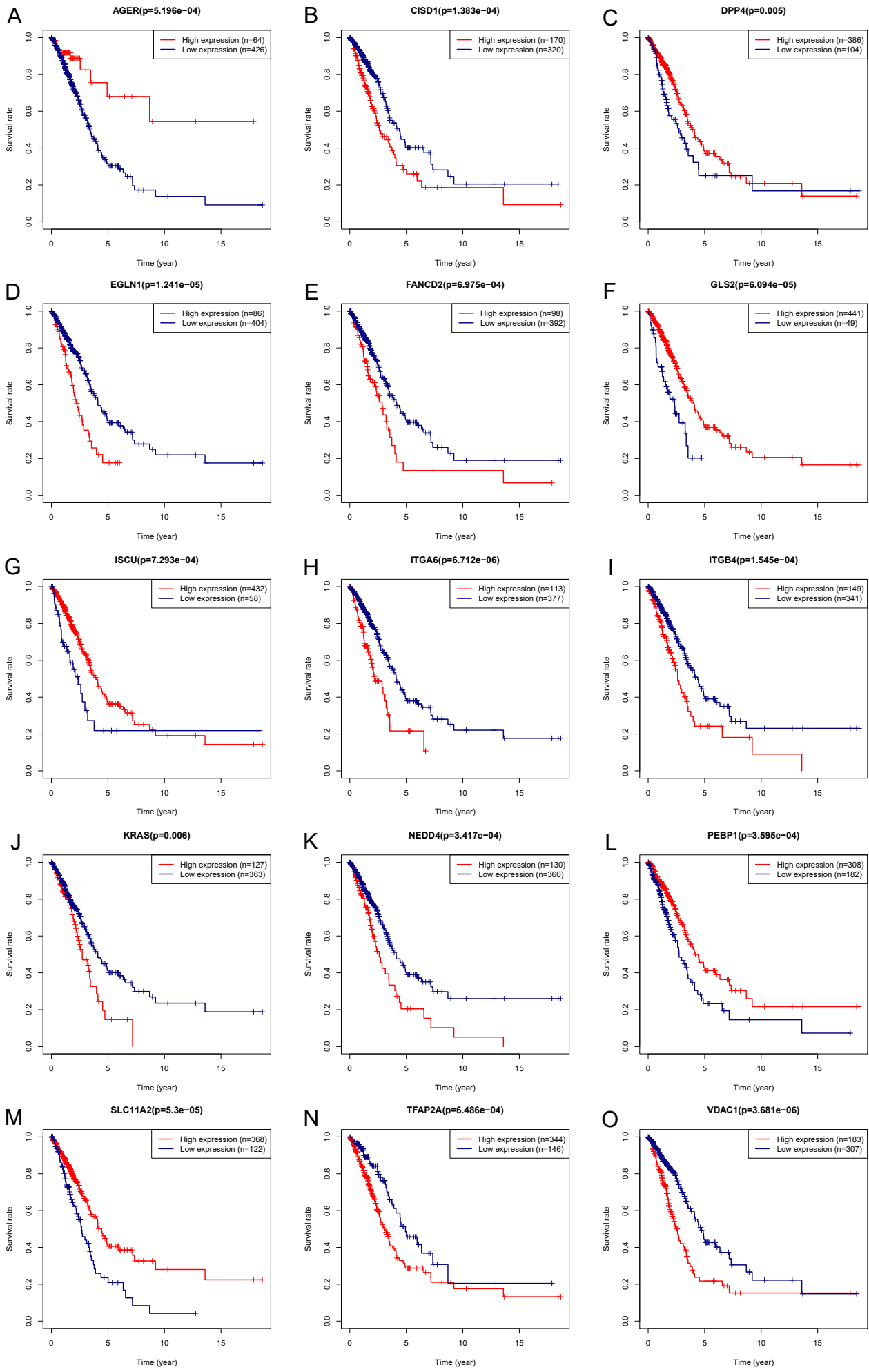

Supplement: Supplemental Information 13 [file peerj-09-11687-s013.pdf]
